# Heterogeneous Integration of Atomically‐Thin Indium Tungsten Oxide Transistors for Low‐Power 3D Monolithic Complementary Inverter

**DOI:** 10.1002/advs.202205481

**Published:** 2023-01-19

**Authors:** Zhen‐Hao Li, Tsung‐Che Chiang, Po‐Yi Kuo, Chun‐Hao Tu, Yue Kuo, Po‐Tsun Liu

**Affiliations:** ^1^ Department of Photonics, College of Electrical and Computer Engineering National Yang Ming Chiao Tung University Hsinchu 30010 Taiwan; ^2^ Department of Electronic Engineering National Chin‐Yi University of Technology Taichung 411030 Taiwan; ^3^ Department of Chemical Engineering Texas A&M University College Station TX 77843‐3127 USA

**Keywords:** atomically‐thin a‐IWO channel, complementary field‐effect transistors, inverter, monolithic 3D‐IC, thin film transistors, 2D materials‐like, vertically‐stacked heterogeneous integration

## Abstract

In this work, the authors demonstrate a novel vertically‐stacked thin film transistor (TFT) architecture for heterogeneously complementary inverter applications, composed of p‐channel polycrystalline silicon (poly‐Si) and n‐channel amorphous indium tungsten oxide (a‐IWO), with a low footprint than planar structure. The a‐IWO TFT with channel thickness of approximately 3‐4 atomic layers exhibits high mobility of 24 cm^2^ V^−1^ s^−1^, near ideally subthreshold swing of 63 mV dec^−1^, low leakage current below 10^−13^ A, high on/off current ratio of larger than 10^9^, extremely small hysteresis of 0 mV, low contact resistance of 0.44 kΩ‐µm, and high stability after encapsulating a passivation layer. The electrical characteristics of n‐channel a‐IWO TFT are well‐matched with p‐channel poly‐Si TFT for superior complementary metal–oxide‐semiconductor technology applications. The inverter can exhibit a high voltage gain of 152 V V^−1^ at low supply voltage of 1.5 V. The noise margin can be up to 80% of supply voltage and perform the symmetrical window. The pico‐watt static power consumption inverter is achieved by the wide energy bandgap of a‐IWO channel and atomically‐thin channel. The vertically‐stacked complementary field‐effect transistors (CFET) with high energy‐efficiency can increase the circuit density in a chip to conform the development of next‐generation semiconductor technology.

## Graphical abstract

The 3D monolithic heterogeneous complementary field‐effect transistor (CFET) integrated with polycrystalline silicon (Poly‐Si) and atomically‐thin amorphous indium tungsten oxide (a‐IWO) TFTs for a vertically stack architecture realizes high voltage gain and low‐power logic circuit, showing the high potential to meet the requirements of next‐generation IC technology with a tiny footprint and extremely high chip density.

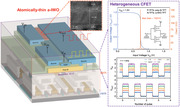

## 1 Introduction

**Figure 1:**
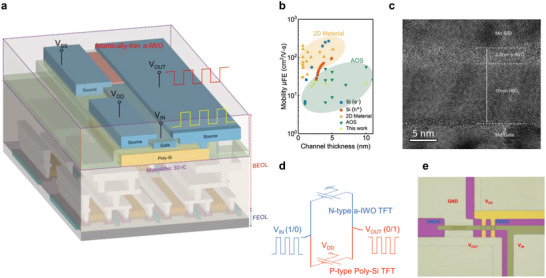
a) Structure schematic of vertical‐stacked heterogeneous complementary field effect transistors (CFET) integrated by thin film transistors technology for the back‐end of line (BEOL)‐compatible monolithic 3D‐ICs application. b) The comparison between channel thickness and field effect mobility in the different material, such as amorphous oxide semiconductor (AOS), 2D material, and single crystalline silicon.^[^ ^22^, ^23^, ^24^, ^25^, ^26^, ^27^, ^28^, ^29^, ^30^, ^31^, ^32^, ^33^, ^34^, ^35^, ^36^, ^37^, ^38^, ^39^, ^40^, ^41^, ^42^ ^]^ c) The transmission electron microscope (TEM) image of cross‐section in atomically‐thin amorphous indium tungsten oxide (a‐IWO) TFTs. d) The inverter equivalent circuit is composed of poly‐Si TFTs and a‐IWO TFTs as the p‐channel TFT (P‐TFT) and n‐channel TFT (N‐TFT), respectively. The input voltage level of high/low (1/0) was converted to the reverse output voltage level of low/high (0/1). e) Top view optical diagram of heterogeneous complementary inverter circuit layout with the common gate and common drain, applying the input voltage ( *V*_IN_ ) and output voltage (*V*_OUT_), respectively. The source of p‐channel and n‐channel TFTs apply the supply voltage (*V*_DD_) and ground (GND), respectively.

The monolithic 3D integrated circuits (M3D‐ICs) technology is promising to overcome area constraints and move toward more‐than‐Moore's law.^[^ ^1^, ^2^ ^]^ In the past, the single‐crystalline silicon‐based complementary metal–oxide‐semiconductor (CMOS) technology was utilized in different logic circuit architectures for different applications, such as Internet of Things, Artificial Intelligence, and 5G communications.^[^ ^3^ ^]^ This was because thin‐film transistors (TFTs) can be fabricated with low thermal budget, which is compatible with M3D technology in the back‐end of line (BEOL) temperature limitation, that is, 525 °C,^[^ ^4^ ^]^ to avoid the degradation of front‐end of line (FEOL) devices. Furthermore, we demonstrate the vertically‐stacked logic circuit based on the TFTs technology in this work to reduce the footprint of devices and increase the chip density, as shown in **Figure** **1**a. In semiconductor materials, the dispersion of energy band determines the carrier mobility of holes and electrons. Hence, transistors suffer from mismatch issues in electrical properties with the homogeneous semiconductor materials used in logic circuit applications (Figure S1, Supporting Information). The integration of heterogeneous materials can offer more flexibility on comparable electrical properties to achieve the high voltage gain and large noise margin for the integrity of the signal transmission in a complementary inverter. For BEOL compatible materials, the p‐channel polycrystalline silicon (poly‐Si) and n‐channel amorphous oxide semiconductor (AOS) with closer and higher carrier mobility provide a solution for the compatibility issues.^[^ ^5^, ^6^, ^7^, ^8^, ^9^ ^]^ This hybrid integration circuit provides the promising potential in low power dissipation due to its complementary configuration and the low leakage current in wide energy bandgap AOS. The hybrid complementary inverter composed of poly‐Si and amorphous indium zinc gallium oxide (a‐IGZO) had been proposed, performing the high gain and full swing properties.^[^ ^10^ ^]^ However, there exists the channel size mismatch issue due to the low carrier mobility of 13 cm^2^ V^−1^ s^−1^ in a‐IGZO thin films. In addition, the thickness of a‐IGZO was typically required up to several tens of nanometer, and this is not conducive to resolve short channel effect (SCE). The carrier mobility can be enhanced by increasing the dopant concentration of indium oxide owing to electronic configuration of heavy post transition indium metal cation of s‐orbital (5s)^[^ ^11^, ^12^ ^]^ with isotropic behavior.^[^ ^13^ ^]^ However, excess indium oxide dopant would typically exhibit “normally‐on” device characteristics, which are not suitable for low‐power circuit applications. Oxygen annealing or oxygen plasma can be used to convert it to normally‐off characteristics, but electrical degradation and stability issues have been reported elsewhere.^[^ ^14^, ^15^ ^]^ Tungsten metal has a high dissociation energy of metal–oxygen bond^[^ ^16^ ^]^ compared to other metals, such as gallium, aluminum,^[^ ^17^ ^]^ and silicon,^[^ ^18^ ^]^ which can effectively suppress excessive carriers and improve the switching characteristics. Nevertheless, excessive doping of tungsten may cause deterioration of mobility, so only small amounts of tungsten can be doped forming amorphous indium tungsten oxide (a‐IWO) for high‐performance transistors.^[^ ^19^ ^]^ Furthermore, the dopant of tungsten can improve the thermal stability to avoid the influence of the subsequent manufacturing processes.^[^ ^20^ ^]^ Alternatively, ultra nano‐sheet (UNS) architecture with a few atomic layers was utilized to enhance the gate control ability to operate in the fully depleting mode, achieving better subthreshold swing (SS), and inhibiting short channel effect.^[^ ^6^, ^21^, ^22^ ^]^ However, there was a trade‐off between the channel layer thickness and mobility,^[^ ^22^, ^23^, ^24^, ^25^, ^26^, ^27^, ^28^, ^29^, ^30^, ^31^, ^32^, ^33^, ^34^, ^35^, ^36^, ^37^, ^38^, ^39^, ^40^, ^41^, ^42^ ^]^ as shown in Figure 1b. The strong scattering effect would occur causing significant degradation of mobility.^[^ ^43^, ^44^ ^]^ In this work, when the channel of a‐IWO TFTs was scaled down to about ≈three to four atomic layers, the carrier transport behavior was similar to that of 2D materials without obvious degradation in carrier mobility.^[^ ^45^ ^]^ The transmission electron microscope (TEM) image of cross‐section is shown in Figure 1c. Separately, as the p‐channel AOS suffers issues from the high density of subgap traps, and large effective mass resulted from the poor hybridization of 2p‐orbital in valence band maximum (VBM),^[^ ^46^ ^]^ its electrical characteristics cannot match those of the n‐channel oxide, which makes it difficult for the high‐performance digital logic circuit. In this work, p‐channel poly‐Si was used as the underlying devices, and vertically integrated with n‐channel atomically‐thin a‐IWO to realize heterogeneous complementary inverter circuits, as shown in Figure 1d. The top view image of complementary TFT inverter is shown in Figure 1e. In comparison with previous studies with planar structure, the proposed vertically‐stacked complementary field effect transistors (CFET) structure shows the potential in low footprint devices and high‐density circuit for the requirements of next‐generation IC technology. The complementary inverter with superior and symmetric electrical characteristics can achieve high voltage gain of 152 V V^−1^, large noise margin window, and low power consumption at supply voltage (*V*_DD_) 1.5 V. All fabrication processes in this work have high stability and uniformity compatible with the current IC industry, demonstrating a highly promising technique for the emerging M3D‐ICs.

## 2 Results and Discussion

### 2.1 N‐Channel Atomically‐Thin a‐IWO TFTs

**Figure 2:**
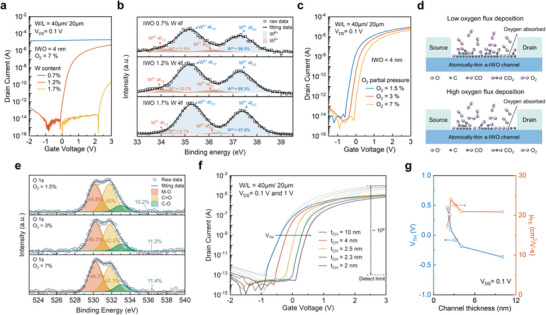
The a) transfer curve and b) X‐ray photoelectron spectroscopy (XPS) W 4f spectra of n‐channel amorphous indium oxide (a‐In_2_O_3_) TFTs doped with different tungsten doping concentration of 0.7%, 1.2% and 1.7%, respectively. The W 4f peak can be resolved into W^6+^ and W^5+^, represented in blue and red, respectively. The c) transfer curve and d) The schematic reaction between C—O related species and oxygen at the high and low oxygen flux deposition, respectively. e) The XPS O 1s spectra of 1.2% tungsten doped a‐In_2_O_3_ surface deposited by different partial pressure of oxygen from 1.5% to 7%. The O 1s is composed of metal–oxygen bond (M—O), carbon=oxygen (C=O), and carbon—oxygen bond (C—O). f) The transfer curve of a‐IWO TFTs with scaling channel thickness from 10 to 2 nm at *V*_DS_ = 0.1 V and 1 V. g) The threshold voltage (*V*_TH_) (left) and field effect mobility (*µ*_FE_) (right) in a‐IWO TFTs as function of channel thickness.

**Figure 3:**
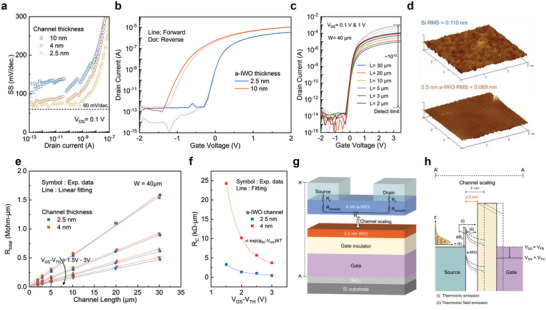
a) The subthreshold swing (SS) of a‐IWO TFT versus drain current for channel thickness of 10, 4, and 2.5 nm at *V*_DS_ = 0.1 V. b) Hysteresis loop curve in a‐IWO channel thickness of 10 and 2.5 nm. c)The transfer curves of a‐IWO TFTs with different channel lengths ranging from 30 to 2 µm at *V*_DS_ = 0.1 and 1 V. d) Comparison of roughness between Si wafer and 2.5 nm a‐IWO measured by atomic force microscope (AFM) image. e) The contact resistance (*R*_C_) extraction by transmission line method (TLM) for 4 nm and 2.5 nm a‐IWO channel, varying overdrive voltage (*V*_OV_ = *V*_GS_−*V*_TH_) from 1.5 to 3 V. f) The *R*c of 2.5 and 4 nm a‐IWO channel versus different *V*_GS_−*V*_TH_ ranging from 1.5 to 3 V, step of 0.5 V. g) The schematic of series resistance in transistors including *R*_C_, parasitic resistance (*R*_parasitic_), and channel resistance (*R*_CH_). h) The energy band diagram of cross‐section from A to A’ for 4 and 2.5 nm. The mechanism of electron transmission is determined by the effect of Schottky barrier bending at *V*_GS_ = *V*_FB_ or *V*_GS_ > *V*_TH_.

In this study, to develop the 3D monolithic architecture, low thermal budget AOS material has been used to avoid the degradation of underlying devices. The development of n‐channel AOS TFT with mobility matching that of the p‐channel poly‐Si TFT (Figure S2, Supporting Information) is critical to achieve excellent voltage transfer characteristics (VTC) of hybrid complementary inverter. In the Si semiconductor, the mobility could be restricted by the carrier concentration because of the coulomb scattering effect.^[^ ^47^ ^]^ In the indium oxide‐based semiconductor, according to the Percolation theory,^[^ ^48^ ^]^ the mobility can be enhanced by increasing the carrier concentration. However, the excess electron carriers in AOS channel could lead to the poor performance in the transistor as above mentioned. Thus, a few amount of tungsten (W) was doped into the indium oxide to improve the switching properties. The variable W content from 0.7% to 1.7% in the a‐IWO channel would significantly affect the transfer characteristic of transistor, as shown in **Figure** **2**a. The device can perform the characteristics of a transistor when the atomic concentration of tungsten is up to 1.2%. In order to further confirm the influence of tungsten dopant on TFT characteristics, the W 4f X‐ray photoelectron spectroscopy (XPS) analysis was executed with different doping concentration, as shown in Figure 2b. The binding energy (284.8) of C 1s was used to calibrate samples. The spectra of W 4f peak was composed of W 4f_7/2_ and W 4f_5//2_, and those peaks also could be resolved into two peaks of five‐ and six‐coordination oxidation states, that is, located at the W^6+^ 4f_7/2_ (35.2 eV), W^6+^ 4f_5//2_ (37.3 eV), W^5+^ 4f_7/2_ (34.0 eV), and W^5+^ 4f_5/2_ (36.1 eV). In detail, signal peaks of W^5+^ and W^6+^ can be attributed to metal–oxygen bond corresponding to WO*_x_* and WO_3_. The ratio of W^5+^ and W^6+^ indicates that the content of the W^6+^ would gradually increase with increasing the tungsten doping concentration. The W^6+^ can capture more oxygen to form WO_3_, which can effectively suppress the excessive oxygen vacancies in a‐IWO channel and achieve the purpose of switching transistors. A higher on‐state current of IWO TFT can be obtained by decreasing the ratio of $\frac{O_{2}}{\mathrm{Ar}+O_{2}}$ from 7% to 1.5%. In general, most studies attribute the increase in conductivity to the environment with low oxygen flux deposition, where there is more oxygen deficiency generation.^[^ ^49^ ^]^ This is also similar to the XPS O 1s results of a‐IWO deconvolution in our study (Figure S4, Supporting Information). However, as the a‐IWO channel thickness is only a few nanometers, the effect of C—O related impurities on the a‐IWO backchannel surface should be considered. The XPS O 1s spectra at the a‐IWO surface was resolved into three peaks, the metal—oxygen bond (M—O) at 530.1 eV, the Carbon=oxygen (C=O) at 531.8 eV, and the carbon‐oxygen bond (C—O) at 533 eV, respectively, as shown in Figure 2e. As the partial pressure of oxygen decreases, the proportion of C—O related impurities in IWO films increases, and the impurity content is up to 55.2% for the IWO film deposited at 1.5% oxygen flux. The C—O species, which are generated in the manufacturing process, would cause the phenomenon of charge transfer as the fermi‐level modulation.^[^ ^50^ ^]^ The energy states of the C—O related impurities are located below the conduction band, which act like shallow donors to contribute electrons during the transistor device operation.^[^ ^51^ ^]^ In addition, the oxygen adsorbed at the back channel reacts with electrons from the a‐IWO channel layer to form O^−^, leading to a lower conductivity of the channel. This phenomenon could be suppressed by the C—O related impurities present on the surface of the a‐IWO back channel, where oxygen reacts with carbon to form C—O species, resulting in the release of electrons from the adsorbed O^−^.^[^ ^52^ ^]^ The reaction schematic is shown in Figure 2d. The above mentioned reasons may be responsible for the excellent conductivity of a‐IWO films deposited at low oxygen flux. Furthermore, it is noteworthy that the off‐current of these oxide TFTs is lower compared to Silicon metal–oxide‐semiconductor field effect transistors (Si MOSFETs), staying below 10^−13^A, regardless of O_2_ concentration, making them more suitable for low‐power applications. This is attributed to the wide band gaps of most oxide semiconductors, which effectively suppress thermally excited carriers (Figure S5, Supporting Information). Both ultraviolet photoelectron spectroscopy (UPS) and optical absorption measurements can also be used to observe the existence of additional density of states (DOS) above the valence band (E_V_), which can effectively suppress the generation of holes in the valence band and reduce the leakage current during off‐state operation.^[^ ^53^ ^]^ Similarly, the TFT deposited from 1.5% O_2_ with high carrier concentration would cause a relatively negative threshold voltage (*V*_TH_). In order to realize the aims of low‐power consumption, the current must be reduced at gate to source voltage less than zero (*V*_GS_ < 0) region. Figure 2f shows the transfer curves of a‐IWO TFTs, where the a‐IWO channel thickness is several atomic layer thickness, ranging from 10 to 2 nm, demonstrating that a more positive *V*_TH_ can be achieved due to the reduction of electron accumulation on channel surface, as shown in Figure 2g. *V*_TH_ in this work was determined from the transfer curve at a constant current of channel width‐to‐length *W*/*L* × 10^−9^ A. The reason is that the electrons in the channel are likely to be depleted easily by atomically‐thin channel structure. However, continued shrinking of the channel thickness also increases the channel resistance, resulting in a decrease in the “on‐state” drain current. Therefore, the mobility of a‐IWO TFT with a 2 nm channel thickness will drop significantly to the 17 cm^2^ V^−1^ s^−1^, making it more difficult to match with p‐TFTs. In this study, the optimized TFT characteristics, that is, field‐effect mobility (*µ*_FE_) of 24 cm^2^ V^−1^ s^−1^, on/off current ratio of 10^9^ at *V*_DS_ = 1 V and SS_min_ ≈ 63 mv dec^−1^, were achieved with the 2.5 nm‐thick a‐IWO channel. Interestingly, as the thickness continued to increase, the field‐effect carrier mobility would saturate because too many carriers accumulated on the channel surface, causing strong scattering between electrons, resulting in a carrier mobility that could not rise with increasing thickness. This trend is different from traditional 3D semiconductor materials. In addition, the atomically thin a‐IWO channel of 2.5 nm also could achieve a near ideal SS for the low supply voltage device originating from the enhancement of gate controllability, as shown in **Figure** **3**a. A cross‐sectional TEM image of 2.5 nm a‐IWO TFT shows the excellent interface between the a‐ IWO channel and HfO_2_ gate dielectric (see Figure 1c). As a result, the extremely small hysteresis loop closed to 0 mV is achieved by the 2.5 nm atomically‐thin a‐IWO TFT, as shown in Figure 3b. The a‐IWO channel thickness also has the influence on the window of the hysteresis loop. The hysteresis loop attributed by the electron capture defects, which are attributed to the electron trapping in defect generated by ion bombardment during the sputtering deposition process, lead to the creation of oxygen interstitials in the deep‐level state.^[^ ^15^ ^]^ The 10 nm‐thick a‐IWO requires a longer deposition time, having more defects present in the channel, and results in a larger *V*_TH_ shift to 0.1 V. These also contribute to the high stability of the atomic‐thin 2.5 nm a‐IWO TFT under the positive bias stress measurement (Figure S6, Supporting Information). The transfer characteristics of IWO TFT with different channel lengths ranging from 30 to 2 µm were shown in Figure 3c. It can be observed that the increase in on‐current is proportional to the decrease in channel length. In addition, the *I*_OFF_ does not change significantly and exhibits extremely low leakage current below the detect limit of measurement (10^−13^A). In the case of the IWO TFT with a channel length of 2 µm, the *I*_ON_/*I*_OFF_ ratio can be ≈10^10^. The atomic force microscopy (AFM) image of the 2.5 nm‐thick a‐IWO layer in Figure 3d displays an extremely low root mean square (RMS) roughness of 0.069 nm. The Dimension Edge AFM used in this study contains a noise floor of 0.05 nm. This result is smaller than the ion radius of 0.07 nm, and it is concluded that there could be measurement uncertainty. Nevertheless, these indicate that the smooth morphology of a‐IWO channel layer can significantly reduce the roughness scattering effect at the interface between the gate insulator and channel layer, and improve the fabrication reliability. The contact resistance (*R*_C_) of a‐IWO TFTs is extracted by transmission line method (TLM),^[^ ^54^ ^]^ as shown in Figure 3e. Figure 3f illustrates that the *R*_C_ of 2.5 nm and 4 nm channel thickness versus different *V*_GS_−*V*_TH_ range from 1.5 to 3 V, in the step of 0.5 V. The contact resistance of Si MOSFET is typically increased by channel thickness thinning due to its parasitic resistance (*R*_parasitic_) generated from the source/drain (S/D) extension region^[^ ^55^ ^]^ and the diminishment of diffusion length (*L*_T_).^[^ ^56^, ^57^ ^]^ In this work; however, it was observed that the *R*_C_ in the a‐IWO TFT reduced with the thinning of the channel layer thickness. It may be contributed by the following two reasons: i) the reduction of channel *R*_parasitic_ from the transistor structure, and ii) the gate‐induced Schottky barrier height (SBH) lowering. First of all, the a‐IWO TFT is used by a bottom‐gate inverted‐staggered structure, where a *R*_parasitic_ exists in the channel segment overlapping between the S/D electrode and gate‐electrode control region, as shown in Figure 3g. The *R*_parasitic_, which is involved in the extraction of the *R*_C_, perpendicular to the channel is proportional to the channel thickness, leading to a decreased *R*_C_ as the channel thickness shrinks. In addition, an exponential drop in the contact resistance can be observed regardless of the channel thickness. Namely, the contact resistance is not only dependent on the Schottky barrier height (*ϕ*_B0_ = *ϕ*_m_ − *χ*), the subtraction of work function of metal (*ϕ*_m_), and the electron affinity of semiconductor (*χ*), but also relates to surface potential (*ϕ*_s_). As the *V*_GS_ is larger than *V*_TH_ (*V*_GS_> *V*_TH_), the electrons would accumulate at the surface to form a channel, and the energy band at the semiconductor surface would be bent.^[^ ^58^, ^59^ ^]^ The thickness of electron accumulation layer is determined by the bending distance. The energy band diagram schematic of the bottom‐gate inverted‐staggered a‐IWO TFT in the A to A' region is shown in Figure 2h. When the IWO channel thickness is reduced to less than the electron accumulation layer thickness (≈1×),^[^ ^60^ ^]^ the gate electric field will further affect the SBH, resulting in the barrier lowering effect for the enhancement of thermionic field emission (TFE).^[^ ^47^ ^]^ In particular, when *V*_GS_ is much larger than *V*_TH_ (*V*_GS_ >> *V*_TH_), the SBH is pulled down dramatically and the electrons will more easily inject into IWO channel from the source electrode, which assists the ohmic contact formation. The relationship of tunneling current density follows $J=J_{0}e^{\left(-\frac{q(\phi_{B0}-\Delta \phi_{B})}{kT}\right)}\left(e^{\left(\frac{qV_{DS}}{nkT}\right)}-1\right)$, where *J*_0_ is associated with Richardson constant at room temperature, q elementary charge, k Boltzmann constant, *T* absolute temperature, and Δ*ϕ*_B_ value of barrier lowering depending on the gate voltage. The contact resistance is dependent on the following equation $Rc={\left(\frac{dJ}{dV_{DS}}\right)}_{V_{DS}=0}^{-1}=J_{0}^{*}e^{\left(\frac{q(\phi_{B0}-\Delta \phi_{B})}{kT}\right)}$.^[^ ^61^ ^]^ Thus, the increase of *V*_GS_ makes the barrier lowering and leads to the exponential reduction of contact resistance. Both of the 2.5 nm (red line) and 4 nm (blue line) IWO layers are thinner than the electron accumulation layer thickness; so, the contact resistance is highly dependent on the gate electrical field. With the thinner channel thickness, the effect of SBH lowering becomes more obvious and the smaller contact resistance of 0.44 kΩ‐µm at *V*_GS_ = 3 V can be obtained. The contact resistance of 2.5 nm atomically‐thin a‐IWO and 2D materials is compared as a function of channel thickness (Figure S7, Supporting Information). The 2D materials can be isolated few layers or even a single monolayer but suffer the issue of large contact resistance, resulting from metal‐induced gap states (MIGS) and the interface barrier between the 2D semiconductor and 3D metal bulk.^[^ ^59^ ^]^ Even if the 2D semiconductor material is heavily doped (>10^13^ cm^−2^) for the purpose of ohmic contact, the transistor would exhibit “normally‐on” characteristics^[^ ^62^, ^63^ ^]^ and cause severe power loss. In summary, the atomically‐thin a‐IWO TFT with several atomic thicknesses of 2.5 nm can perform well in all electrical characteristics, namely mobility, SS, off‐state current, and exhibit the ultralow contact resistance applied for the short‐channel transistor device, compared to other semiconductors. Furthermore, normally‐off devices can be obtained through tungsten doping concentration, oxygen partial pressure, and channel thickness to improve the energy efficiency during circuit operation.

### 2.2 Monolithic Vertically‐Stacked Heterogeneous Complementary Inverters

**Figure 4:**
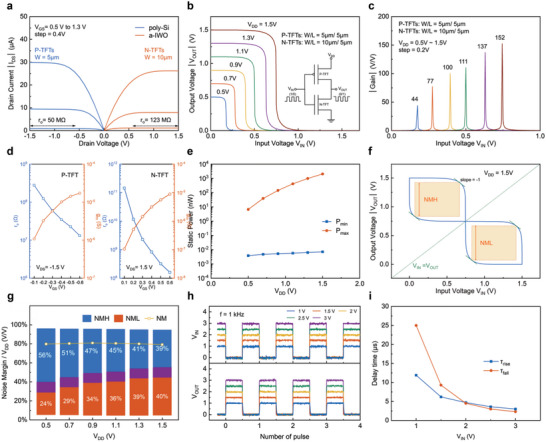
Vertically‐stacked hybrid integration complementary inverter circuit based on TFTs. a) The symmetric output curve (*I*_DS_−*V*_DS_) of P‐TFTs and N‐TFT at *V*_GS_ of 0.5 V, 1.1 V 1.3 V, and with channel width‐to‐length *W*/*L* = 5 µm/5 µm and *W*/*L* = 10 µm/5 µm. b) The voltage transfer curve of inverters varying the *V*_DD_ from 0.5 to 1.5 V. c) The extraction of voltage gain (|d*V*_IN_/d*V*_OUT_|) corresponding to different *V*_DD_. d) The extraction of output resistance (*r*_o_) and transfer conductance (*g*_m_) as function of *V*_GS_ in P‐TFT and N‐TFT. e) The static power consumption of inverter as a function of *V*_DD_ from 0.5 to 1.5 V. f) The extraction of noise margin by mirror coupling of VTC curve. g) The noise margin high (NMH) and noise magin low (NML) at *V*_DD_ from 0.5 to 1.5 V. h) The dynamic characteristic waveform of inverter at *V*_DD_ from 1 to 3 V. i) The delay time of inverter extracted by output waveform at *V*_DD_ from the 1 to 3 V.

As mentioned previously, the n‐channel a‐IWO TFT is promising CFET integrated with p‐channel poly‐Si to achieve the hybrid complementary inverter circuit in the BEOL‐compatible 3D‐IC applications. The footprint is critical for high‐density integrated circuits to break through the physical limitations of size scaling. The vertical stack architecture is an effective arrangement for the reduction of device footprint. After the poly‐Si TFT processes were completed, a‐IWO TFT with the low thermal budget (<250 °C) was stacked subsequently on the poly‐Si TFT as the upper layer device. The output curves of the P‐TFT and N‐TFT are well‐matched, as shown in **Figure** **4**a. The identically high *r*_o_ of 50 and 123 MΩ can be obtained for the P‐TFT and the N‐TFT, respectively. The channel length modulation effect is suppressed in both P‐TFT and N‐TFT, assisting in improvement of intrinsic gain (*A*_i_ = *g*_m_ *r*_o_) for a single transistor, where g_m_ is tconductance and r_o_ is ouput resistance. Furthermore, with the operation of the inverter at the transient region between high level and low level, the P‐TFT and N‐TFT are operated at the saturation region, while the high output impedance can also enhance the inverter to access a steeper VTC curve. On the other hand, the a‐IWO channel with high carrier density was hardly depleted for suppression of channel length modulation, causing the N‐TFT with larger *r*_o_. Figure 4b shows the VTC curves of the hybrid complementary inverter with different *V*_DD_ ranging from 0.5 to 1.5 V. The channel width‐to‐length ratio of P‐TFT and N‐TFT is 5 µm/5 µm and 10 µm/5 µm, respectively. The output voltage (*V*_OUT_) of logic “1” is equal to the *V*_DD_, while the output voltage of logic “0” is equal to the ground, clearly presenting the excellent inverter with rail‐to‐rail full swing characteristics. As shown in Figure 4c, the voltage gain extracted from *V*_OUT_ derivation of input voltage (*V*_IN_) (|d*V*_OUT_/d*V*_IN_|) increases linearly from 44 V/V to 152 V/V when the *V*_DD_ rises from 0.5 to 1.5 V. This contributes to the robust intrinsic gain of the P‐TFT and N‐TFT without degradation, depicted in Figure 4d. It is also worth noting that the intrinsic gain of a‐IWO TFTs can be greater than 1000, which is much larger than that of Si MOSFET.^[^ ^6^ ^]^ Furthermore, the off‐current of the TFT made of the wide band gap a‐IWO is much lower than those of Si MOSFETs and transistors made of 2D materials or other compound semiconductors. The static powers of inverters are illustrated in Figure 4e. In this work, the proposed hybrid TFTs‐based complementary inverter can only consume several pico‐watt (pW) when operating at high/low level state region. Thus, the integration of poly‐Si TFT and a‐IWO TFT for the high‐performance inverter has been achieved successfully, exhibiting extremely high voltage gain with small *V*_DD_ and ultra‐low power. It is promising for the low‐power IC applications. The extraction of noise margin was executed by the mirror coupling of voltage transfer relationship, as shown in Figure 4f. It shows the symmetrical noise margin window at *V*_DD_ = 1.5 V, and both of the noise margin high (NM_H_) and the noise margin low (NM_L_) are 0.6 V. The statistic schematic of noise margin proportions with different *V*_DD_ are plotted in Figure 4g. The ideal symmetry and remarkable noise margin window are clearly observed in the proposed hybrid complementary TFT inverters. The dynamic performance of the inverter with different pulse height from 1 to 3 V is also shown in Figure 4h. The rising time (*τ*_rise_) and falling time (*τ*_fall_) was measured at *V*_IN_ = *V*_DD_ from 1 to 3 V and step = 0.5 V. The high current density is expected to boost the operating speed and consequently reduce the delay time, as shown in Figure 4i. The delay time can be diminished close to several microseconds as the *V*_GS_ increases. The operation frequency is limited approximately in sub‐megahertz, attributed to the large size of TFT devices with 5 µm channel length, limited by the lithographic capabilities in this work, and it can be greatly promoted by the channel length scaling in the future work. These results highlight the fact that the proposed inverter is compatible for low‐power applications, which offers excellent voltage gain at small *V*_DD_ and exhibits a significantly low static power under a normalized voltage gain condition, in comparison with previous reports on heterogeneous inverter (Figure S8, Supporting Information).

## 3 Conclusion

We have demonstrated, for the first time, a vertically stacked hybrid complementary inverter circuit with the combination of LC‐NILC p‐channel poly‐Si TFT and n‐channel atomically‐thin a‐IWO TFT. In comparison with the previously proposed planar structure, the 3D monolithic vertically‐stacked TFT can reduce the device footprint and further increase the chip density of the M3D‐IC. All the processes are compatible with the BEOL process. The implementation of the a‐IWO channel with serval atomic layers (2L‐4L) can effectively enhance the gate control ability of transistors to fully deplete the highly conductive channel to be off‐state for low power consumption. Meanwhile, the remarkable electrical characteristics are achieved, with high field‐effect mobility of 24 cm^2^ V^−1^ s^−1^, and excellent SS of 64 mV dec^−1^, suitable for the heterogeneous CFET inverter with symmetric and preferable voltage transfer curves. The state‐of‐the‐art voltage transfer characteristics of the inverter with a sharp transition level and high voltage gain of 152 V V^−1^ can be obtained at the small *V*_DD_. The low static power dissipation at high/low level and transition level is pico‐watt and nano‐watt, respectively, which is compatible for low‐power circuit applications. Furthermore, the large noise margin window effectively enhances the circuit stability as the inverter is under operation. The proposed heterogeneous integration complementary TFTs‐based inverter with high energy efficiency has great potential in next‐generation semiconductor technology of M3D‐ICs.

## 4 Experimental Section

### Devices Fabrication

The hybrid complementary TFTs were integrated by the use of low metal comtamination nickel‐induced lateral crystallization (LC‐NILC) poly‐Si P‐TFT and a‐IWO N‐TFT. The vertically‐ staked device architecture was implemented by the completion of P‐TFT, followed by the stacking N‐TFT. First, a 50 nm‐thick amorphous silicon (a‐Si) was deposited on a layer of SiO_2_ film acting as the BEOL interlayer. The a‐Si film was patterned as P‐TFT active region by inductively coupled plasmaetch process. The windows for NILC process were patterned; and then, nickel seed layer was deposited on the a‐Si layer by the electron‐beam evaporation. The lift‐off process was used to form the asymmetric Ni seeding window. Afterward, the nickel–silicide was formed at the interface between Ni and a‐Si by annealing process, and then, the redundant Ni regions were removed to avoid the Ni contamination. The layer of a‐Si film converted to the poly‐Si film below 500 °C when the Ni atoms diffused to the source region from the drain side, which was carried out at a thermal furnace in N_2_ ambient. A counterpart of the poly‐Si TFT device fabricated by solid phase crystallization (SPC) method at 600 °C was also used as a reference sample for comparison. After the formation of dummy gate, the channel region was defined. The implantation of BF_2_ was carried out to form source/drain regions of poly‐Si P‐TFT devices. The dummy gate was removed sequentially before 15 nm‐thick HfO_2_ was deposited as the gate insulator of P‐TFT by atomic layer deposition (ALD). A layer of Mo film was deposited as the common‐gate electrode of the inverter. In this step, the P‐TFTs were completed, and the fabrication of N‐TFTs was followed up. A 10 nm‐thick HfO_2_ was deposited by ALD process acting as the gate insulator of N‐TFT. The a‐IWO channel layer of N‐TFT was then deposited by radio frequency (RF) sputtering. By moderating oxygen partial and the channel thickness of a‐IWO layer, the semiconductor properties could be optimized with 2.5 nm thickness. Before the Mo deposition as the source/drain electrodes of N‐TFT, the contact holes for source/drain regions were formed by dry etching process. Last, the heterogeneous CFET was completed and the channel width‐to‐length ratios of P‐TFT and N‐TFT was 10 µm/5 µm and 20 µm/5 µm, respectively.

## Conflict of Interest

The authors declare no conflict of interest.

## Supporting information

Supporting Information

## Data Availability

The data that support the findings of this study are available from the corresponding author upon reasonable request.
