# Supplementary material for: Heterogeneous Integration of Atomically‐Thin Indium Tungsten Oxide Transistors for Low‐Power 3D Monolithic Complementary Inverter
Source: Adv Sci (Weinh). 2023 Jan 19;10(9):2205481. doi: 10.1002/advs.202205481 (PMC10037976; doi:10.1002/advs.202205481)
Supplement: Supplementary file 1 — Supporting Information [file ADVS-10-2205481-s001.pdf]

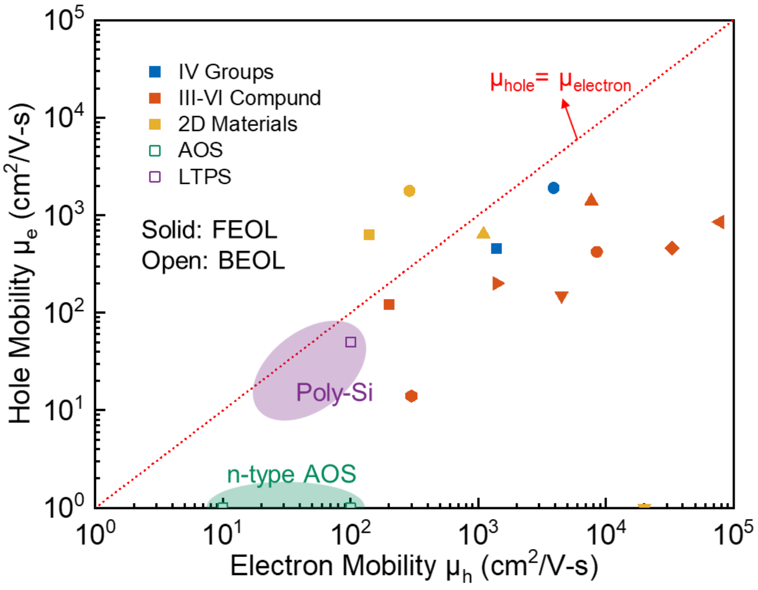

Figure S1 | Comparison of hole carrier mobility and electron carrier mobility between different semiconductor materials. In numerous semiconductor materials such as IV groups semiconductors, III-VI compound semiconductors[1], and 2D materials[2-4], there are different carrier mobilities between hole and electron because of the different effective masses, capable of calculating from the energy band diagram. The equation of carrier mobility can be determined by $\mu = q\tau_c/m^*$, where q is the elementary charge, τ_c the mean free path of carrier, and m* the effective mass. Thus, the mismatch of electrical properties for the homogeneous semiconductor material is a critical issue for circuit design applications. In this work, the hybrid configuration was proposed for the device integration. In addition, the thin film transistor (TFT) technology was adopted for the back-end-of-line (BEOL) compatible process and sequential vertically-stacked structure due to its low thermal budget. The p-channel low-temperature polycrystalline silicon (LTPS) and n-channel amorphous oxide semiconductor (AOS) behave the similar carrier mobility, and thereby the mismatch of electrical characteristics can be relieved to achieve the high-performance logic circuit applications.

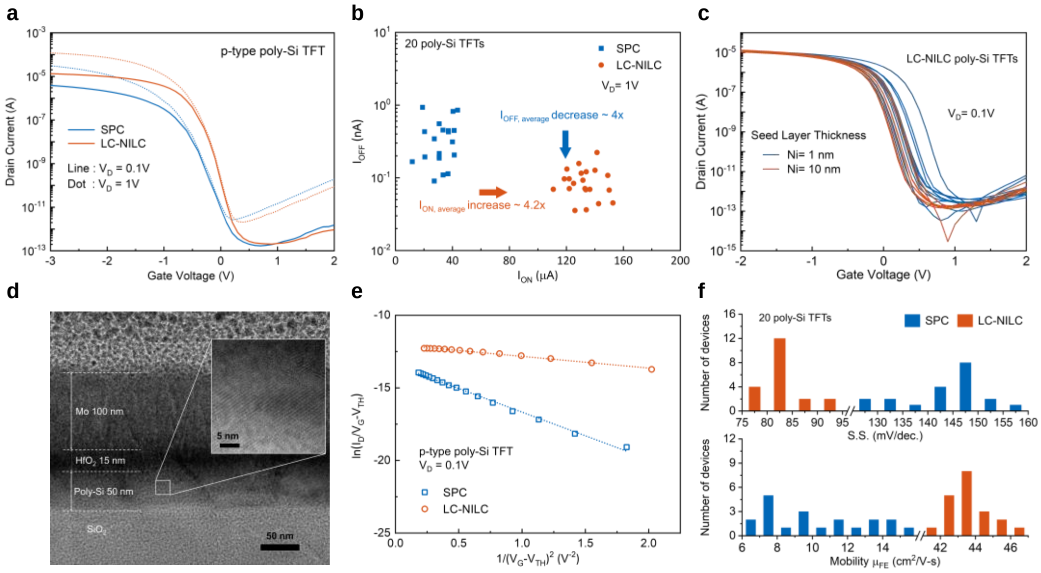

Figure S2 | Low contamination nickel-induced laterally crystallization p-channel poly-Si TFTs. The conventional solid-phase crystallization (SPC) method can be used to transform the amorphous silicon (a-Si) into poly-Si with small grain size at temperatures above 600˚C. In contrast, poly-Si film with large grains can be formed from a-Si below 500˚C using the nickel-induced crystalline (NIC) method for monolithic 3D-ICs. However, NIC and NILC method usually encounters the problem of leaving Ni residues in the poly-Si thin film during the Ni diffusion process. In this work, the low-contamination nickel-induced lateral crystallization (LC-NILC) poly-Si was proposed for the suppression of Ni metal contaminant to apply in p-type TFTs of inverter[5]. Figure S2 (a) shows transfer curves of p-channel poly-Si TFTs crystallized by LC-NILC method and SPC method. The LC-NILC poly-Si TFT exhibits better electrical characteristics than the SPC poly-Si TFT with field effect mobility (μ_{FE}) of 43 cm^2/V-s and subthreshold swing of 84 mV/dec. for the LC-NILC poly-Si is better than that of SPC poly-Si with 10 cm^2/V-s and 145 mV/dec, respectively. As shown in Figure S2 (b), it indicates that the relationship between I_{ON} and I_{OFF} for the p-channel poly-Si TFTs with the crystallization methods of LC-NILC and SPC V_{DS} = 1V in measurements of twenty devices. The average I_{ON} of the former is 4.2 times larger, and the average I_{OFF} is 4 times smaller than that of the latter. In addition, the thickness of nickel seed layer is the critical condition for the quality of LC-NILC poly-Si, which determines the diffusion amount of nickel metal, and resultantly tuning the threshold voltage (V_{TH}) of LC-NILC poly-Si TFT for inverter circuit application, as shown in Figure S2 (c). As the nickel diffused laterally from the drain electrode to the source electrode, part of the nickel atoms were retained in the poly-Si channel. The metallic nickel residues would form charged trap centers at the interface between the channel and gate insulator and lead to the positive shift of V_{TH}. The optimum electrical

properties and uniformity of the LC-NILC poly-Si are realized by using a 10 nm-thick nickel seed layer. Figure S2 (d) shows the TEM image of the LC-NILC poly-Si, indicating that the Si atoms the single crystalline-like phase in local channel regions. Figure S2 (e) shows the plot of $\ln[I_D/(V_{GS}-V_{FB})]$ versus $1/(V_{GS}-V_{FB})^2$ curves of p-channel poly-Si TFTs by the Levinson and Proano method, where flat band voltage (V_{FB}) is defined as the V_{TH}. The trap-state density resulted from poly-Si grain boundary can be determined from the square root of the slope, i.e., $N_{trap}=(C_{OX}\sqrt{slope})/q$ where C_{OX} is the oxide capacitor per unit area, and q is the magnitude of elementary charge. The trap state densities of the LC-NILC and SPC poly-Si TFT of 4.95×10^{12} cm^{-2} and 9.99×10^{12} cm^{-2}, respectively. This is consistent with their electrical characteristics. The electrical uniformity has always been a major concern for the poly-Si TFT devices. Figure 2(f) shows the histograms of S.S. and μ_{FE} distributions of 20 p-channel poly-Si TFTs made with LC-NILC and SPC methods. The LC-NILC poly-Si TFTs exhibit better and narrower distributions of electrical characteristics than the SPC poly-Si TFTs.

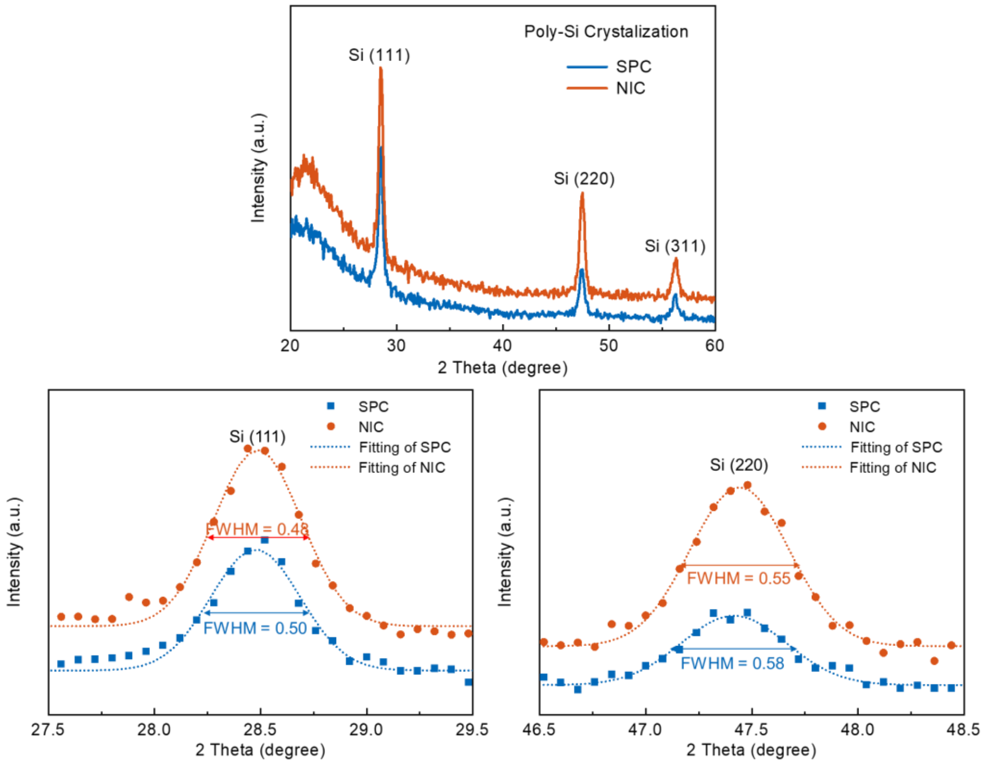

Figure S3 | X-ray diffraction peaks of poly-Si films for orientation (111), (220) and (311) with solid-phase crystallization (SPC) and nickel induced crystallization (NIC) methods. Polycrystalline silicon films are composed of independent crystalline grains so that numerous defect centers exit at the grain boundaries and cause leakage currents, degrading transistor performance. The XRD peak signals of (111) and (220) exhibit the largest intensity at $2\theta = 28.4^{\circ}$ and $47.5\ ^{\circ}$ in this poly-Si thin film, respectively. Furthermore, it is clearly observed from the smaller full-width at half maximum (FWHM) of XRD peak that the poly-Si exhibits great crystallinity in the (111) and (220). The FWHM is obtained by a Gaussian function fitting and the grain size (τ) can be extracted from Scherrer equation as followed,

$$\tau = \frac{K\lambda}{\beta\cos\theta}$$

where K is the crystallite-shape factor, λ is the wavelength of the X-ray, β is the FWHM of the XRD peak and θ is the Bragg angle. The NIC poly-Si exhibits excellent uniformity and large grain size than SPC poly-Si.

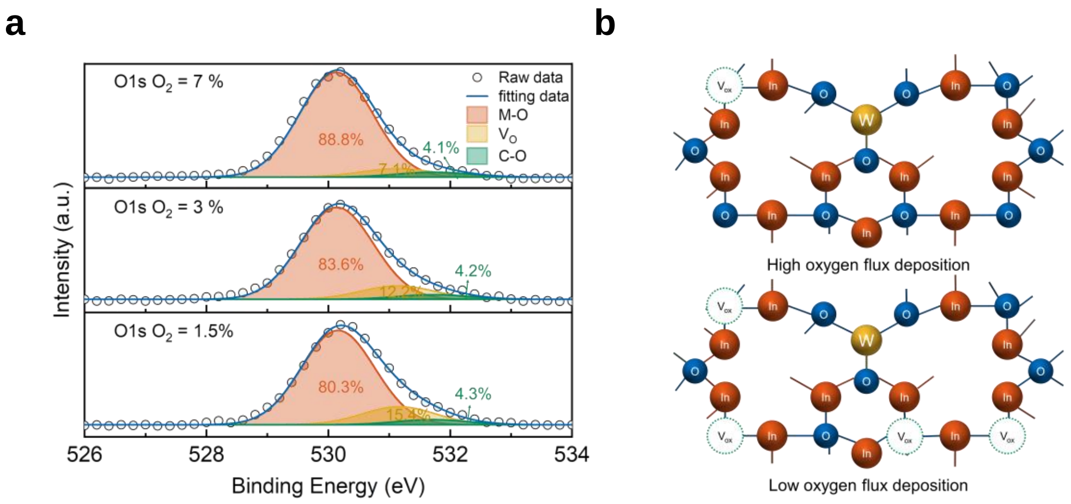

Figure S4 | XPS O 1s spectra of 1.2% tungsten doped a-In_2O_3 channel, which was deposited by the different partial pressure of oxygen in a mixture of O_2/Ar gases from 1.5% to 7%. The O 1s is composed of metal-oxygen bond (M-O), oxygen vacancy (Vo) and carbon-oxygen bond (C-O). The XPS of O 1s spectra was resolved into three peaks, the metal-oxygen bond (M-O) at 530.2 eV, the oxygen vacancy (V_O) at 531.2 eV, and the carbon-oxygen bond (C-O) at 531.8 eV, respectively. The oxygen vacancy is well known as the donor in oxide semiconductor materials because its energy state level exists below the conduction band. Namely, the oxygen vacancies of the a-IWO film deposited at high-oxygen-flow are compensated by the incoming oxygen during the sputtering process leading to the decreases of a-IWO conductivity.

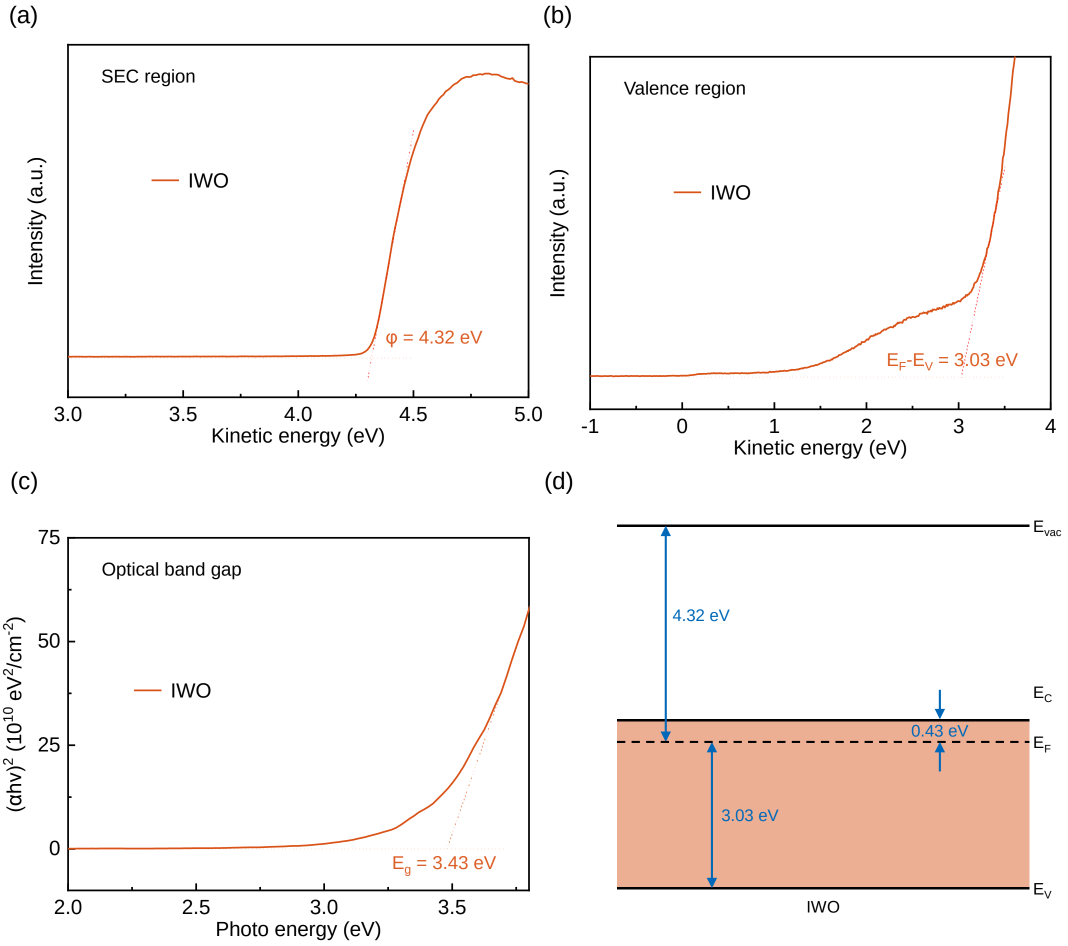

Figure S5 | The Ultraviolet Photoelectron Spectroscopy (UPS) spectra of (a) secondary electron cutoff (SEC) region and (b) valence band region. (c) The extraction of optical band gap by Tauc plot method. (d) The energy band structure schematic of a-IWO thin film. The energy band diagram schematic of a-IWO thin film was extracted by the UPS and transmittance spectra. The SEC region and valence band region in UPS analysis indicate that the work function (φ) was 4.32 eV and E_F-E_V of 3.03 eV, respectively. The energy band gap (E_g) of 3.44 eV can effectively suppress the leakage current of a-IWO TFTs for low power consumption.

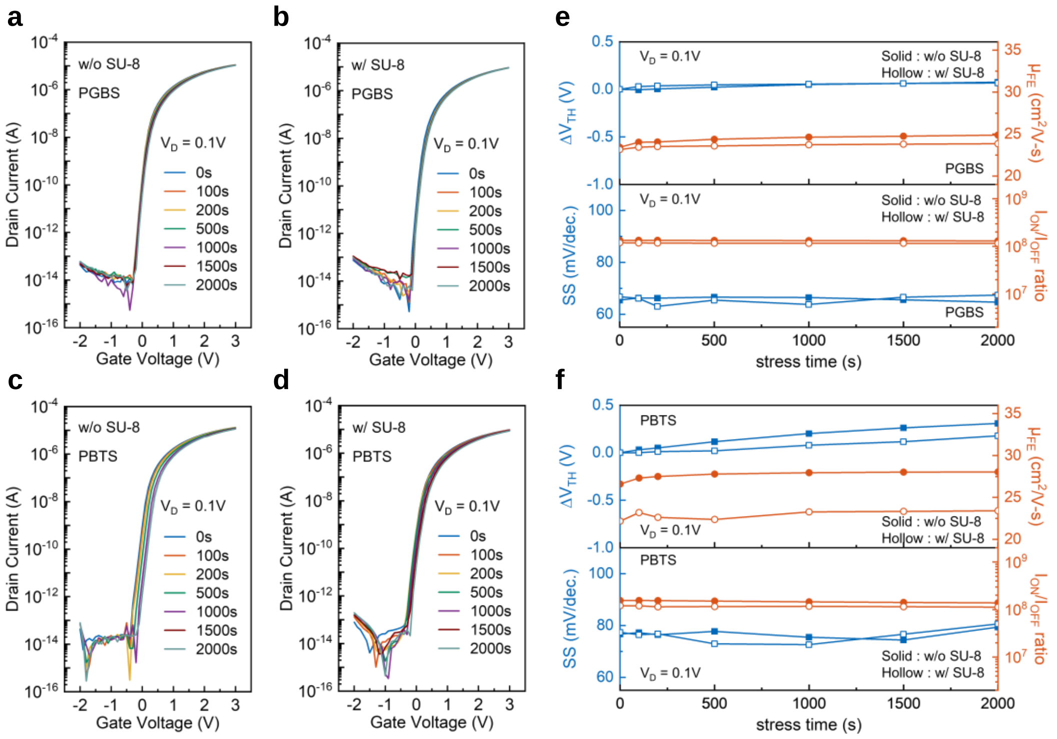

Figure S6 | The stress measurement of 2.5 nm a-IWO TFTs without and with SU-8 passivation layer in (a)(b) PGBS (c)(d) PBTS at 60˚C, respectively, under gate electrical field 2 MV/cm for 2000s. The data extraction of (e) PGBS and (f) PBTS measurement. Amorphous oxide semiconductor (AOS) TFTs are often affected by interfacial defects and ambient oxygen under prolonged operation, resulting in electrical degradation, such as threshold voltage (V_{TH}) shift, and mobility drop. A passivation layer is needed for the AOS channel area to resist the influence of the environment. SU-8 is an organic epoxy-based polymer with excellent chemical stability, suitable for use as a passivation layer[6]. Figure S5 (a)-(d) show the stress measurement of the 2.5nm-thick IWO TFT without and with SU-8 passivation layer under positive gate bias stress (PGBS) and positive bias thermal stress (PBTS) at 60˚C, with a gate electric field of 2MV/cm for the 2000s, respectively. The IWO TFT device maintained excellent characteristics after the coating process with the SU-8 layer. Furthermore, no significant degradation in carrier mobility, SS, and I_{ON}/I_{OFF} ratio was observed for both TFT devices. That is, even without the channel passivation layer, as shown in Figure S5 (e)-(d), respectively. The IWO TFT exhibits high reliability, especially in PGBS measurement, which is attributed to the superior interface of the atomically thin a-IWO channel. After encapsulating the passivation layer, the stability of IWO TFT can be improved with a V_{TH} shift of 0.18 V. These are all attributed to the fact that $O_2 + e^- \rightarrow 2O^-$ reaction can be effectively suppressed due to the enhanced gate control capability of atomically-thin a-IWO [7], preventing the

oxygen atoms adsorbed in the IWO channel from trapping electrons and resultantly forming O_2^-.

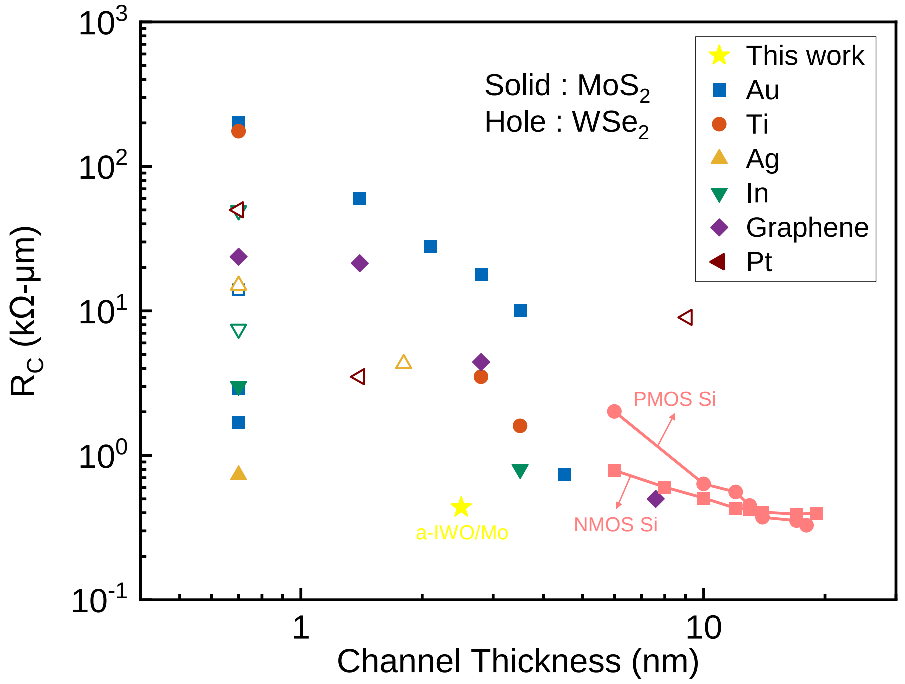

Figure S7 | The benchmark of contact resistance in this work, 2D-material and FinFET Si. In order to suppress punch through currents between S/D electrodes, the transistors must be executed with an ultra-thin channel thickness. In the case of nanometer channel thickness, the contact resistance will increase significantly, affecting the electrical characteristics of the transistors. For example, two-dimensional semiconductors are widely studied because they can effectively miniaturize channel thickness to the atomic level. However, they suffer the issue of high contact resistance due to the high Schottky barrier and metal induced gap states (MIGS), resulting the poor electrical characteristics. In this work, the thickness of the proposed a-IWO channel can be reduced to 2.5 nm, which is approximately 3 or 4 atomic layers. The UNS TFTs can achieve the low contact resistance of about 0.4 kΩ-μm since the vertical electrical field induce the Schottky barrier lowering and narrowing as mentioned in the article. In addition, IWO materials offer excellent uniformity, superior electrical properties, high process compatibility and low thermal budgets, making them suitable for 3D IC applications. The benchmark of UNS a-IWO TFTs were shown in the Figure S5 and Table. I.

Table S1| Benchmark of UNS a-IWO TFTs technology.

channel	Contact metal	Channel thickness (nm)	Contact resistance (kΩ-µm)	Channel deposition	Channel thermal budget (˚C)	Ref.
**a-IWO**	Mo	2.5	0.4	PVD	room temp.	This work
**MoS_2**	Au	4.5	0.74	Exfoliated	-	[8]
	Au	0.7	200	Exfoliated	-	[9]
		2.8	18			
	Au	0.7	1.7	CVD	930	[10]
	Au/Ti/Au	0.7	2.9	CVD	930	[11]
	Ti/Au	0.7	175	CVD	850	[12]
	Ti	2.8	3.5	Exfoliated	-	[13]
		3.5	1.6			
	Ag/Au	0.7	0.73	CVD	850	[14]
	In	0.7	3	CVD	870	[15]
		3.5	0.8			
	graphene	0.7	23.7	Exfoliated	-	[16]
		1.4	21.4			
		2.8	4.4			
	graphene/Ni	7.6	0.5	Exfoliated	-	[13]
**WSe_2**	Au	0.7	14	Exfoliated		[17]
	Ti	0.7	3.6×10^4	Exfoliated	-	[18]
	Ag	0.7	15			
	In	0.7	7.5			
	WO_3/Ag	1.8	4.3	Exfoliated	-	[19]
	Graphene/Ti	0.7	49.4	CVD	-	[20]
	Pt/Au	1.4	3.5	Exfoliated	-	[21]
		9.1	9			
**n-Si**	NiSi	6	0.79	SOI	-	[22]
		10	0.51			
		12	0.43			
		17	0.39			
**p-Si**	NiSi	6	2.0			
		10	0.63			
		12	0.56			
		17	0.35			

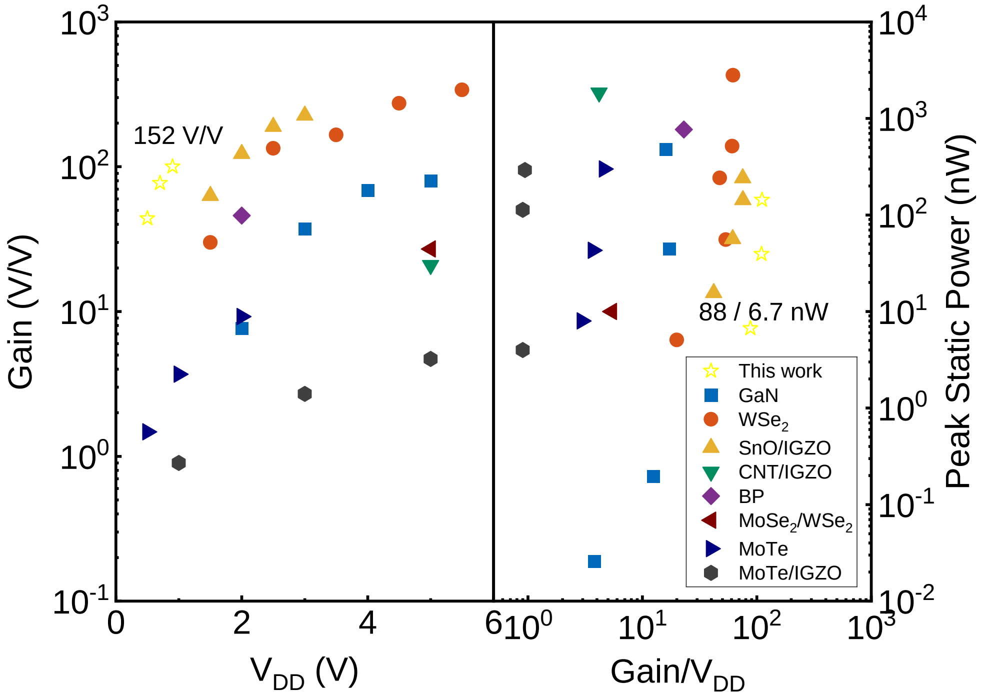

Figure S8 | (a) State-of-the-art voltage gain for inverter plotted as function of V_{GS} and (b) the peak static power-loss as function of Gain/V_{DD}. The hybrid complementary TFTs can achieve extremely high voltage gain with low supply voltage, which contributes to the high gain of transistors, especially the n-channel a-IWO TFTs. In addition, the minimum and maximum static power dissipation was limited at pico-watt level and nano-watt level, respectively. On this basis, these hybrid inverters can provide better energy efficiency than previously reported for inverters, such as 2D materials, oxide semiconductor and III-V group semiconductor. That is, it can consume less power to obtain a relatively high gain. The comparison of inverter performance in this work with previous reports were shown in Table II

Table S2 | The comparison of inverter performance in this work with previous reports.

p-channel	n-channel	Structure	V_{DD} (V)	Gain (V/V)	P_{max}/Gain (nW)	P_{min} (pW)	Ref.
Poly-Si	a-IWO	Vertical	0.5	44	0.15	3.84	This work
			0.7	77	0.51	4.72	
			0.9	100	1.4	5.26	
			1.5	152	13.5	7.13	
GaN	GaN	planar	2	8	0.004	8.73	[23]
			5	80	6.25	53.63	
WSe_2	WSe_2	planar	1.5	30	0.17	-	[17]
			5.5	340	8.26	-	
SnO	IGZO	planar	1.5	63	0.25	2	[24]
			3	226	1.07	8100	
CNT	IGZO	planar	5	21	89	950	[25]
BP	BP	planar	2	46	17	215000	[26]
MoS_2	WSe_2	planar	5	27	0.4	4.06	[27]
MoTe	MoTe	planar	0.5	1.5	5.42	500	[28]
			2	9.3	32.49	18000	
MoTe	IGZO	planar	1	0.9	4.44	-	[29]
			5	4.7	62.13	-	

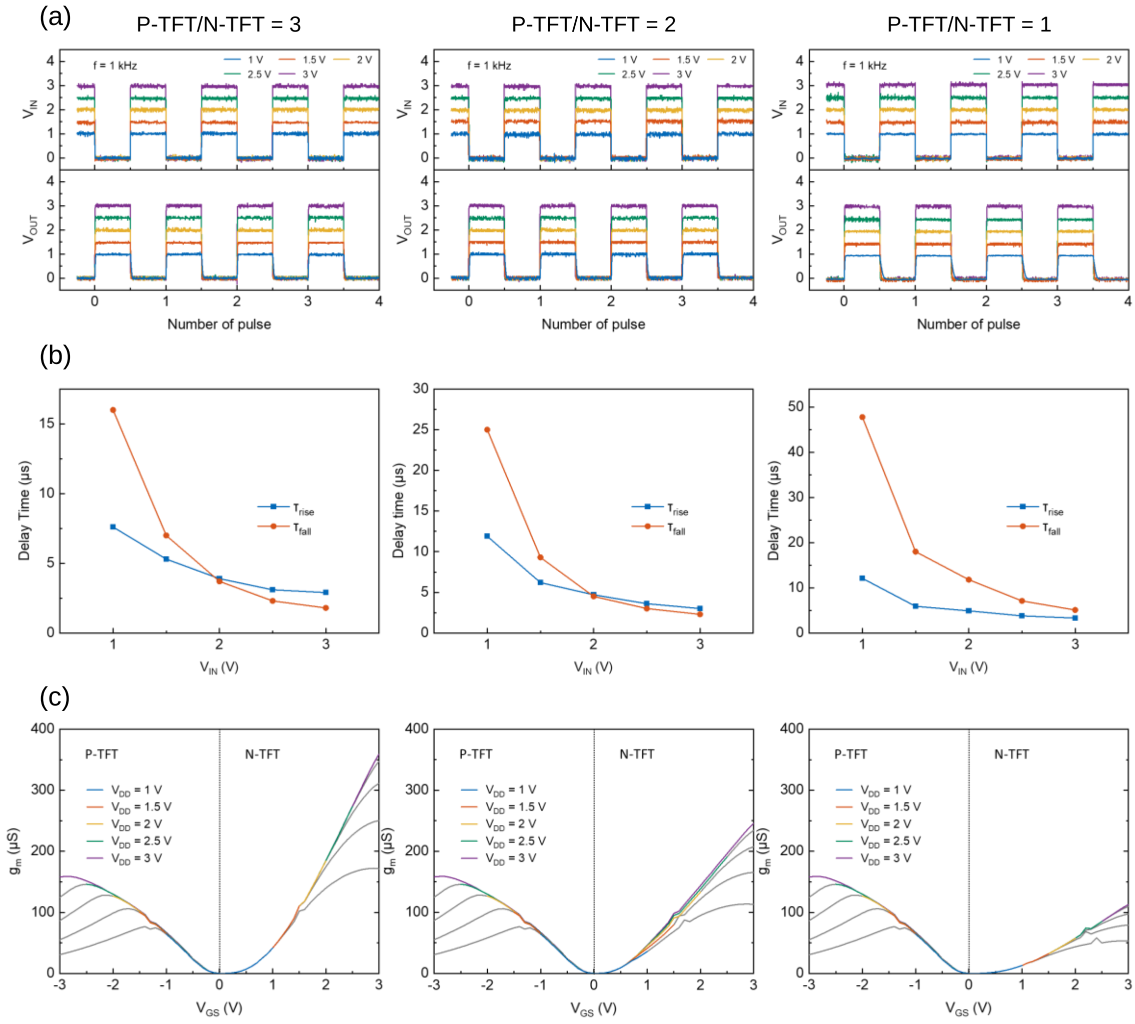

Figure S9 | (a) The waveform of input voltage (V_{IN}) and output voltage (V_{OUT}) with various V_{DD} at driving frequency of 1k Hz. (b) The rising time (τ_{rise}) and falling time (τ_{fall}) of hybrid complementary inverter as a function of V_{DD} from the 1 V to 3 V. (c) The extraction of transfer conductance (g_m) in P-TFT and N-TFT with various V_{DD} from 1 V to 3V. The figures from the left to the right represent the different W/L ratios ranging from 3 to 1 of P-TFT and N-TFT. A signal with a continuous pulse height equal to V_{DD} was applied to the V_{IN} of the hybrid complementary inverter through the Keithley 4225-PMU ultra-fast I-V module, and the V_{DD} is the DC bias. The V_{OUT} waveform of the inverter was measured by an oscilloscope at different pulse height of V_{IN} from 1 V to 3 V, and extracted the τ_{rise} and τ_{fall}, which are defined as the time from 10% to 90% of the change between the high level and low level stage. It cloud be observed that the fast driving speed was achieved as the V_{DD} increased due to the high driving current of transistors. The falling time (τ_{fall}) was close to the rising time (τ_{rise}) at V_{IN} = 2 V, but the τ_{fall} was lower than τ_{rise} as V_{IN} over 2 V resulting from the g_m value of TFTs. The g_m of P-TFT was larger than that of N-TFT as V_{IN} < 2 V, closed to N-TFT as V_{IN} = 2 V, and it was

smaller than that of N-TFT as $V_{IN} > 2$ V. The superior performance of the inverter circuit is attributed to the AOS carrier transfer mechanism with a percolation effect in the high V_{GS} electrical field region, which can avoid the g_m degradation from coulomb scattering. The smallest value of the τ_{rise} and τ_{fall} was approximately 2.9 μs and 1.8 μs at $V_{DD} = 3$ V, respectively. These values were not small enough compared to the traditional CMOS inverter circuit due to the larger channel size (L= 5 μm) and the magnitude of delay time of V_{IN} (100 ns). The performance of the hybrid complementary inverter can be effectively accelerated by reducing the channel size of TFT device to avoid signal distortion under the high frequency operation.

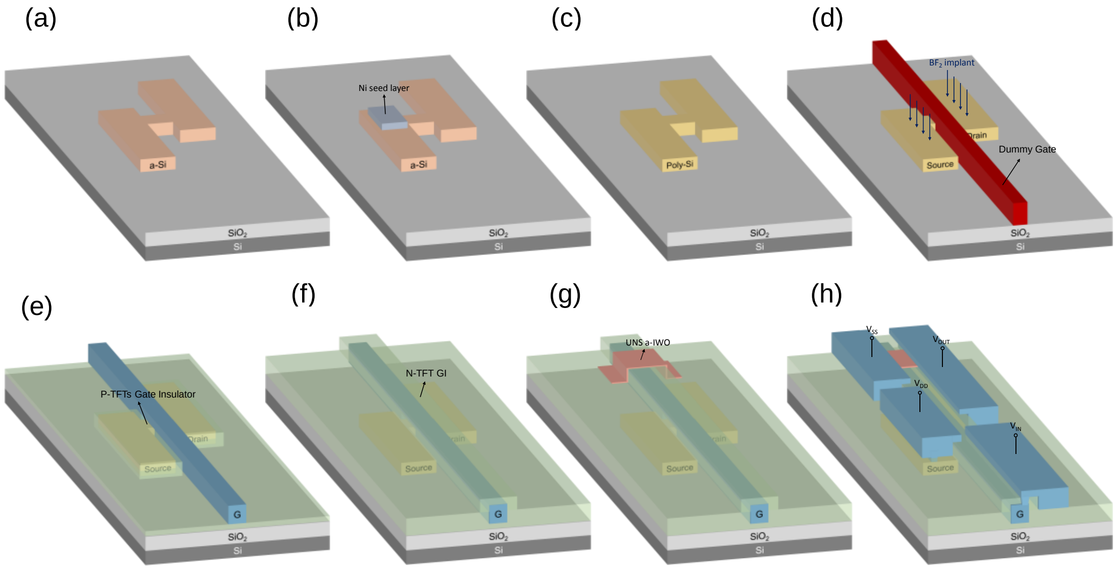

Figure S10 | Schematics of manufacture flow of hybrid complementary TFT-based inverter circuit with vertically-stacked architecture. (a) Firstly, the 50 nm-thick amorphous silicon (a-Si) was deposited on a layer of SiO_2 film and defined as P-TFT active region by inductively coupled plasma (ICP) etching process. **(b)** The Ni metal was deposited on the a-Si layer by electron-beam evaporation for forming the asymmetric NILC seed layer window. After formation of the nickel-silicide, the redundant Ni regions were removed to reduce the Ni contamination. **(c)** The Ni-induced poly-Si layer was formed by the thermal annealing process at a thermal furnace at 500˚C in N_2 ambient. **(d)** The source/drain regions of P-TFT were defined by BF_2 ion implantation process, after the formation of the patterned photoresist of dummy gate. **(e)** It was followed to remove the dummy gate photoresist and then 15nm-thick HfO_2 was deposited as the gate insulator (GI) of P-TFT by plasma enhance atomic layer deposition (PEALD). A layer of Mo metal was deposited by DC sputtering and patterned as inverter common-gate electrode. **(f)** In similar, a 10 nm-thick HfO_2 layer was formed by PEALD process as the GI of N-TFT. **(g)** The a-IWO film layer was then deposited by radio frequency (RF) sputter at room temperature acting as the channel layer of N-TFT. **(h)** Finally, the vertically-stacked hybrid complementary TFT-based inverter was completed after the metallization process.

Reference

[1] O. Moutanabbir, U. Gösele, *Annual Review of Materials Research* **2010**, *40* (1), 469.

[2] Y. Ma, A. Kuc, Y. Jing, P. Philipsen, T. Heine, *Angewandte Chemie International Edition* **2017**, *56* (34), 10214.

[3] J. Qiao, X. Kong, Z.-X. Hu, F. Yang, W. Ji, *Nature communications* **2014**, *5* (1), 1.

[4] K. S. Novoselov, A. K. Geim, S. V. Morozov, D.-e. Jiang, Y. Zhang, S. V. Dubonos, I. V. Grigorieva, A. A. Firsov, *science* **2004**, *306* (5696), 666.

[5] P.-Y. Kuo, S.-C. Lo, H.-H. Wei, P.-T. Liu, *IEEE Journal of the Electron Devices Society* **2020**, *8*, 1317.

[6] A. Olziersky, P. Barquinha, A. Vila, L. Pereira, G. Gonçalves, E. Fortunato, R. Martins, J. R. Morante, *Journal of Applied Physics* **2010**, *108* (6), 064505.

[7] J. K. Jeong, H. Won Yang, J. H. Jeong, Y.-G. Mo, H. D. Kim, *Applied Physics Letters* **2008**, *93* (12), 123508.

[8] C. D. English, G. Shine, V. E. Dorgan, K. C. Saraswat, E. Pop, *Nano letters* **2016**, *16* (6), 3824.

[9] S.-L. Li, K. Komatsu, S. Nakaharai, Y.-F. Lin, M. Yamamoto, X. Duan, K. Tsukagoshi, *ACS nano* **2014**, *8* (12), 12836.

[10] C. D. English, K. K. Smithe, R. L. Xu, E. Pop, in *2016 IEEE International Electron Devices Meeting (IEDM)* IEEE, **2016**, 5.6. 1-5.6. 4.

[11] N. Li, Q. Wang, C. Shen, Z. Wei, H. Yu, J. Zhao, X. Lu, G. Wang, C. He, L. Xie, *Nature Electronics* **2020**, *3* (11), 711.

[12] H. Liu, M. Si, S. Najmaei, A. T. Neal, Y. Du, P. M. Ajayan, J. Lou, P. D. Ye, *Nano letters* **2013**, *13* (6), 2640.

[13] Y. Liu, J. Guo, Y. Wu, E. Zhu, N. O. Weiss, Q. He, H. Wu, H.-C. Cheng, Y. Xu, I. Shakir, *Nano letters* **2016**, *16* (10), 6337.

[14] K. K. Smithe, S. V. Suryavanshi, M. Muñoz Rojo, A. D. Tedjarati, E. Pop, *ACS nano* **2017**, *11* (8), 8456.

[15] Y. Wang, J. C. Kim, R. J. Wu, J. Martinez, X. Song, J. Yang, F. Zhao, A. Mkhoyan, H. Y. Jeong, M. Chhowalla, *Nature* **2019**, *568* (7750), 70.

[16] X. Cui, G.-H. Lee, Y. D. Kim, G. Arefe, P. Y. Huang, C.-H. Lee, D. A. Chenet, X. Zhang, L. Wang, F. Ye, *Nature nanotechnology* **2015**, *10* (6), 534.

[17] L. Kong, X. Zhang, Q. Tao, M. Zhang, W. Dang, Z. Li, L. Feng, L. Liao, X. Duan, Y. Liu, *Nature communications* **2020**, *11* (1), 1.

[18] W. Liu, J. Kang, D. Sarkar, Y. Khatami, D. Jena, K. Banerjee, *Nano letters* **2013**, *13* (5), 1983.

[19] M. Sivan, Y. Li, H. Veluri, Y. Zhao, B. Tang, X. Wang, E. Zamburg, J. F. Leong, J.

X. Niu, U. Chand, *Nature communications* **2019**, *10* (1), 1.

[20] H.-L. Tang, M.-H. Chiu, C.-C. Tseng, S.-H. Yang, K.-J. Hou, S.-Y. Wei, J.-K. Huang, Y.-F. Lin, C.-H. Lien, L.-J. Li, *ACS nano* **2017**, *11* (12), 12817.

[21] Y. Jung, M. S. Choi, A. Nipane, A. Borah, B. Kim, A. Zangiabadi, T. Taniguchi, K. Watanabe, W. J. Yoo, J. Hone, *Nature Electronics* **2019**, *2* (5), 187.

[22] M. Van Dal, N. Collaert, G. Doornbos, G. Vellianitis, G. Curatola, B. Pawlak, R. Duffy, C. Jonville, B. Degroote, E. Altamirano, in *2007 IEEE symposium on VLSI technology* IEEE, **2007**, 110-111.

[23] Z. Zheng, L. Zhang, W. Song, S. Feng, H. Xu, J. Sun, S. Yang, T. Chen, J. Wei, K. J. Chen, *Nature Electronics* **2021**, *4* (8), 595.

[24] Y. Yuan, J. Yang, Z. Hu, Y. Li, L. Du, Y. Wang, L. Zhou, Q. Wang, A. Song, Q. Xin, *IEEE Electron Device Letters* **2018**, *39* (11), 1676.

[25] H. Chen, Y. Cao, J. Zhang, C. Zhou, *Nature communications* **2014**, *5* (1), 1.

[26] S. P. Koenig, R. A. Doganov, L. Seixas, A. Carvalho, J. Y. Tan, K. Watanabe, T. Taniguchi, N. Yakovlev, A. H. Castro Neto, B. Ozyilmaz, *Nano Letters* **2016**, *16* (4), 2145.

[27] P. J. Jeon, J. S. Kim, J. Y. Lim, Y. Cho, A. Pezeshki, H. S. Lee, S. Yu, S.-W. Min, S. Im, *ACS applied materials & interfaces* **2015**, *7* (40), 22333.

[28] Y. J. Park, A. K. Katiyar, A. T. Hoang, J. H. Ahn, *Small* **2019**, *15* (28), 1901772.

[29] H. S. Lee, K. Choi, J. S. Kim, S. Yu, K. R. Ko, S. Im, *ACS Applied Materials & Interfaces* **2017**, *9* (18), 15592.
